# IPL-M1 interaction shapes pre-reflective social differentiation in the human action system: new insights from TBS and TMS combined

**DOI:** 10.1038/s41598-018-30480-z

**Published:** 2018-08-10

**Authors:** Luca F. Ticini, Thomas Dolk, Florian Waszak, Simone Schütz-Bosbach

**Affiliations:** 1 Division of Neuroscience and Experimental Psychology, School of Biological Sciences, Faculty of Biology, Medicine and Health, University of Manchester, Manchester Academic Health Science Centre, Manchester, M13 9PL UK; 2 Department of Experimental Psychology, University of Regensburg, Regensburg, Germany; 3 Université Paris Descartes and CNRS, Paris, France; 4 Department of Psychology, Ludwig Maximilians University Munich, München, Germany

## Abstract

The conscious experience of being the author of our own actions is thought to be grounded in pre-reflective and low-level sensorimotor representations of the self as different from the other. It has been suggested that the inferior parietal lobe (IPL) is generally involved in self-other differentiation processes and in providing an explicit sense of action authorship. However, direct evidence for its causal and functional role in distinguishing self-related and other-related sensorimotor representations is lacking. The current study employed theta-burst stimulation (TBS) to condition left IPL’s activity before a social version of the rubber hand illusion led participants to illusorily attribute observed finger movements to their own body. We recorded motor evoked potentials to single-pulse transcranial magnetic stimulation over the primary motor cortex (M1) as proxies of action authorship during action observation. The results showed that in a control condition (intermediate TBS over the left IPL) others’ actions facilitated whereas self-attributed movements inhibited the motor system. Critically, continuous TBS disrupted this mismatch between self and other representations. This outcome provides direct evidence for the IPL’s role in providing fundamental authorship signals for social differentiation in the human action system.

## Introduction

A central debate in the investigation of human behaviour concerns the origins of subjective action states, such as the daily effortless experience of being the author of one’s own movements. Indeed, how and where in the brain this experience is formed and which neural computations support it are still not fully understood. Studies in experimental psychology and cognitive neuroscience have identified a number of distinct dimensions and indicators of human selfhood. On the one hand, a conceptual and reflective form of agency and authorship experience is thought to rely on complex processes of belief formation (see^1–5^). On the other hand, a pre-reflective sensorimotor experience of action authorship — the subject of the present work — is thought to be automatically generated by primary processes of perception-action coupling^6,7^. This hypothesis goes back to classical motor control theories proposing that during self-generated movements, the brain would compare the efferent copy^8^ of the executed motor command with the sensory consequences of the action^6,7,9^. A strong correspondence between the two is associated with the experience that events are self-generated^1,10^, whilst a discrepancy informs the brain that an action is executed by another agent. These by-products of bodily activity are constantly present and may explain why our sense of self persists (the so-called minimal self^11^) even when we are not engaged in explicit reflection and monitoring of movements.

Previously, Schütz-Bosbach and colleagues^12,13^ characterised the proxy measures of this low-level experience of action authorship in the human motor system. In their work, they combined single-pulse transcranial magnetic stimulation (spTMS) with a social version of the ‘rubber hand illusion’ (RHI^14^). During the RHI, the participants observed a model abducting the right index finger while their own hand was hidden from view. The dorsal surface of the model’s (observed) and of the participant’s (occluded) right index fingers were stimulated by either synchronous or asynchronous brush-stroking delivered by two identical small paintbrushes. A successful RHI manipulation resulted in the participant’s experience of ownership of the actions of the model’s hand during synchronous stroking, as assessed through a questionnaire^14^. Therefore, the RHI allowed to compare action authorship when movements, which were physically equivalent, were either illusorily attributed or not attributed to the self^12^. As a sensorimotor marker of action authorship, Schütz-Bosbach and colleagues recorded spTMS-induced motor evoked potentials (MEP) triggered by action observation in the peripheral right hand muscles of the index (involved in the observed action) and little (control) fingers. The results showed that the motor system differentiates between self- and other-attributed actions: while the observation of actions linked to another individual facilitated the motor system, following a mirror-matching mechanism (see also^15^), the observation of actions attributed to the self inhibited it (see^13^).

However, the question of which neurophysiological mechanisms shape these sensorimotor markers of action authorship still remains unanswered. The current study aimed to address this issue by specifically testing the functional contribution of the inferior parietal lobe (IPL).

At a cognitive level, this cortical structure is known to play a key role in the explicit attribution of actions to their agents as well as in the awareness of ones’ own movement execution^16–20^ (for a review, see^21,22^), and in self-other differentiation^23–25^. Moreover, computational models^26,27^ and brain imaging research suggest that this area works as a comparator mechanism (see also^1,27–29^) shaping the feeling of control and causation over an action (the feeling of ‘agency’; e.g.^30–34^) when the predicted and actual sensory consequences of the action correspond.

To date, it is unclear whether the IPL exerts an influence on the pre-reflective experience of action authorship, as measured in the experiments of Schütz-Bosbach and colleagues^12,13^. Two recent studies support the idea that the IPL is directly involved in processing self-related actions at a sensorimotor level. The first study^35^ showed that, during an imitation-inhibition task, facilitation of the right IPL by means of anodal transcranial alternating current stimulation (tDCS) enhanced self-related motor representations measured as spTMS-induced MEPs. The second study^36^ investigated with electroencephalography the motor responses to the observation of hand movements. The result showed that alternating (disruptive) tDCS of the left IPL reduced the activation of self-related motor processes during the observation of hand movements from an egocentric (but not allocentric) viewpoint.

We therefore hypothesised that, if IPL is indeed involved in shaping action authorship at a sensorimotor level, its transient distribution would alter the pattern of MEPs as measured by Schütz-Bosbach and colleagues^12^. In particular, we reasoned that left IPL interference might drop the known inhibitory effects of the IPL on the ipsilateral primary motor cortex^37^, which are likely associated with intra-cortical inhibition and reduced facilitation measured for self-owned actions during the RHI^13^.

To test this, we interfered with IPL’s activity (as a marker of action authorship) before recording amplitude variations of MEPs evoked by applying spTMS to the ipsilateral left primary motor cortex. We targeted the left IPL with trains of offline noninvasive continuous theta-burst-stimulation (cTBS^38^), an established tool to directly test the functional role of brain areas in cognitive processes. In a within-subjects design, we applied intermediate TBS (imTBS) as a control condition to the same area, as it is known to produce no significant excitability changes in the cortex^38–40^. After brain stimulation and during the recordings of MEPs, participants observed the hand of a human model performing abductions with the contralateral right index finger. By means of the RHI^14^, the hand performing these actions was illusorily attributed to the self (during synchronous brush-stroking) or to the model (during asynchronous stroking^12,13^). Summing up, in this study we measured variations of MEP amplitudes triggered by action observation when the perceived movements were execute by a hand attributed to the self or to the other, in conditions in which interferential brain stimulation was or was not applied over the left IPL.

In the control condition (i.e. imTBS), we expected to record smaller MEP amplitudes when the observed actions were illusorily linked to the self (during synchronous stroking) as compared to MEPs associated with actions attributed to another individual (during asynchronous stroking^12,13^). After cTBS, we expected this self-other differentiation to disappear. This result would indicate that action authorship was lost after disruptive stimulation of IPL thereby supporting a causal role of parietal neural computations in shaping action attribution at the sensorimotor level.

## Materials and Methods

### Participants

Sixteen female subjects (26.4 ± 4.3 years old) with no neurological history participated in the study. We determined the required sample size through the G* power software^41^ by setting the expected effect size at 0.41 (estimated from^13^), the significance level at 0.05, and the desired power at 0.95. All participants had normal or corrected-to-normal vision. They were right-handed as assessed by the Edinburgh Handedness Inventory^42^ and naïve with regard to the purpose of the study. The protocol was approved by the ethic committee of the University of Leipzig and written informed consent was requested. The research was performed in accordance with relevant safety guidelines^43^.

### Procedure and experimental design

The experiment followed a within-participants design, with the independent variables STROKING (“Synchronous/Self”, during synchronous stroking, see below, vs. “Asynchronous/Other”, during asynchronous stroking) and TBS (cTBS vs. imTBS). STROKING and TBS were blocked and the order of blocks was counterbalanced across participants (Table 1). Each subject participated to the experiment twice, in two sessions (1st and 2nd day) of either cTBS or imTBS, separated by 7.1 ± 0.77 days.

**Table 1:** Balancing of the experimental sessions per group of participants.

Group	1st day			2nd day		
	TBS stimulation	Induction Block (begins with)	Experimental Block (begins with)	TBS stimulation	Induction Block (begins with)	Experimental Block (begins with)
1	imTBS	Synchronous	Asynchronous	cTBS	Synchronous	Asynchronous
2	imTBS	Asynchronous	Synchronous	cTBS	Asynchronous	Synchronous
3	cTBS	Synchronous	Asynchronous	imTBS	Synchronous	Asynchronous
4	cTBS	Asynchronous	Synchronous	imTBS	Asynchronous	Synchronous

#### The rubber hand illusion

**Figure 1:**
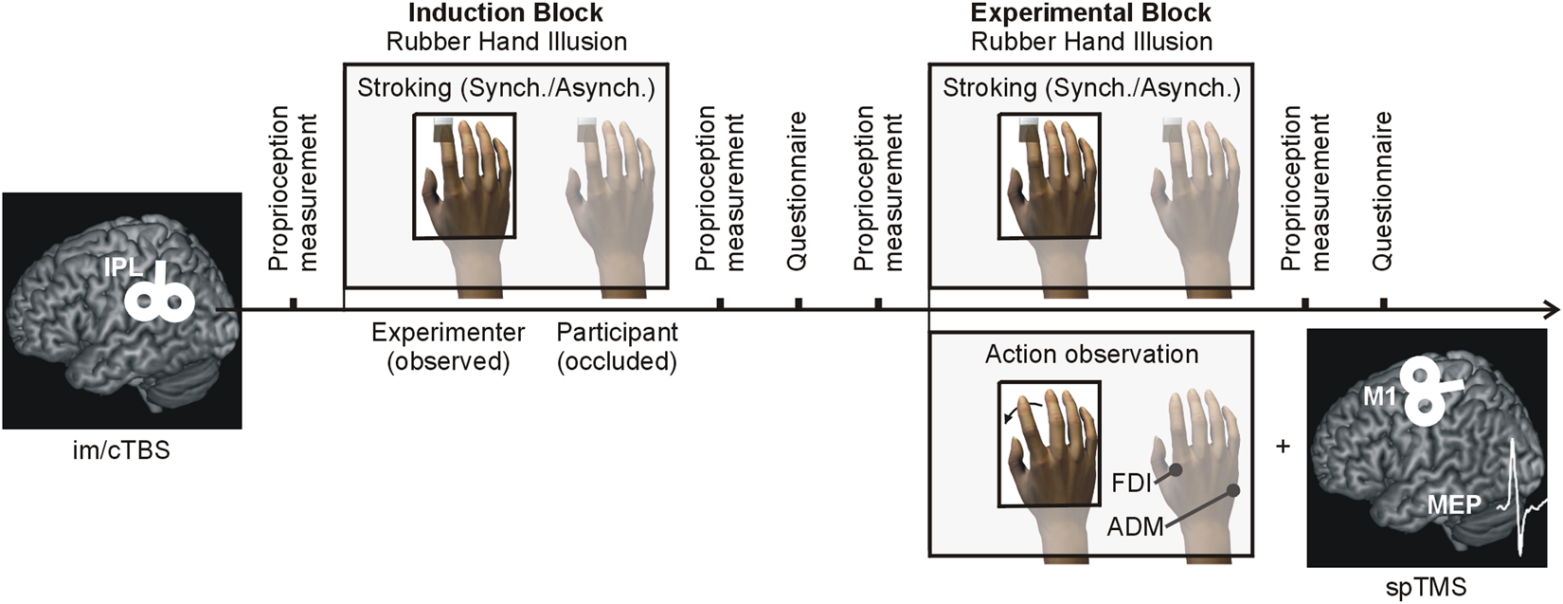
Illustration of the Experiment. Targeting the left inferior parietal lobe (IPL), each of the two days started with the application of either continuous theta-burst stimulation (cTBS) or intermediate (im)TBS (order counterbalanced across participants). In the following Induction and Experimental Blocks, the Rubber Hand Illusion was induced as a consequence of synchronous as compared to asynchronous stroking. The successful induction of the illusion was qualified by proprioceptive measurements prior and after the each block and by an ownership questionnaire after them. In the Experimental Block, participants observed abductions of a model’s right index finger that was illusorily attributed to themselves in the synchronous condition or to the model after asynchronous stroking. At the same time, spTMS-induced MEPs from the right index and the little (i.e., control) finger were recorded. This setup allowed to investigate the modulations of the observer’s motor cortex associated with the observation of actions attributed to the self or to another individual.

We used a modified version of the RHI, as described by Schütz-Bosbach *et al*.^12^, in which the participants illusorily attribute (or not) the hand of a model (a human experimenter) to their own body. As the model was a female and to maintain constant the experimental settings of the RHI (see also below) we chose participants of the same gender. Participants sat in a sound-attenuated dimly lit room keeping their right hand in a pronated position, hidden from view inside a box. They were asked to observe the right hand of the model, sitting on their left side (index-to-index fixed distance of 23 cm^44^), under a surface mounted on top of the box that appeared either as a mirror or as transparent glass, according to computer-controlled illumination. The dorsal surface of the model’s (observed) and of the participant’s (occluded) right index fingers were stimulated with either synchronously or asynchronously stroking, by two identical small paintbrushes mounted on computer-controlled motors (Fig. 1). Both stroke types were of identical duration. Only during synchronous stroking, the strokes applied on the model and on the participant had identical time onset, speed, duration and direction of tactile simulation (from the knuckle to the fingertip or vice-versa). To avoid habituation, strokes’ direction and speed were kept unpredictable by random changes every three seconds. Previous studies showed that only synchronous stroking (i.e., congruent visual and tactile stimulation) induces in participants the feeling that the model’s hand belongs to their own body^14,45,46^. The RHI also keeps the experience of the observed hand identical with respect to viewpoint, morphological features, proprioceptive information and kinaesthetic experience.

In an Induction Block (Fig. 1) the participants observed the model’s relaxed hand, while either synchronous or asynchronous stokes were delivered for three minutes. In a subsequent Experimental Block (EB), one minute of either synchronous or asynchronous stroking was followed by short unpredictable periods of action observation trials (N = 20) in which the participants observed the abduction of the model’s right index finger, while the stroking was stopped (a GO signal was delivered to the model via headphones, and was the last of three auditory signals at 1 Hz). These trials alternated with short periods of stroking (randomised between 4 and 7 seconds).

#### Measures of Body Ownership

When stroking was absent, before and after IB and EB, the surface covering the model’s hand appeared as a mirror (hence the hand was occluded) and the proprioceptive drift measure was taken. The proprioceptive drift indicates quantitatively the displacement of the participant’s felt hand position towards the model’s hand. The participants verbally indicated the perceived location of their right index finger based on the numbers depicted on a ruler reflected on the mirror surface (for more details, see^46^). To avoid response bias, the ruler was always presented with a random offset. After IB and EB, a questionnaire, shortened and translated into a German version of the official RHI questionnaire^14^, assessed ownership of the observed hand. The participants rated the strength of agreement or disagreement with 4 statements: (1) ‘It seemed as if I was feeling the touch of the paintbrush in the location where I saw the other person’s hand touched’; (2) ‘It seemed as if the touch I felt was caused by the paintbrush touching the other person’s hand’; (3) ‘I felt as if he other person’s hand was my hand’. A further statement assessed potential agency experience: (4) ‘I had the feeling to be in control of the other hand’. The rating was conducted on a visual analogue scale from left (0 = ‘completely disagree’) to right (10 = ‘completely agree’). Higher agreement would indicate that the participants experienced the RHI (statements 1 to 3) and felt to be the agent of the observed finger’s abduction (statement 4).

### Transcranial magnetic stimulation

#### Theta-burst-stimulation

At the beginning of each day of recordings, we applied TBS (either cTBS or imTBS^38^) to the EEG-marker position Cp5 (see^47^). Although stereotaxic positioning of the TMS coil provides better accuracy than EEG-guided TMS^47,48^, the 10–20 system is also extensively used. According to Herwig *et al*.^49^, Cp5 stimulation targets the left IPL, comprising the supramarginal (BA 40) and adjacent angular (BA 39) gyri^50^. We applied either cTBS or imTBS as they are known to have different effects on corticospinal excitability: it is believed that cTBS suppresses corticospinal excitability^51^ whilst imTBS produces no excitability changes, thereby it is considered as a reliable control stimulation^38^. Compared to other brain stimulation paradigms, such as 1 Hz repetitive TMS, TBS uses relatively low intensities and it is therefore more tolerated. Moreover, cTBS effects last longer (approx. 30 min.) compared to standard 1 Hz stimulation^52^. In our experiment, TBS was delivered through an air-cooled figure-of-eight coil of 70 cm diameter attached to a Magstim Rapid2 stimulator (The Magstim Company Ltd, Whitland, U.K.). The stimulation pattern consisted of bursts of three pulses at 50 Hz (20 ms between each pulse), which were repeatedly delivered every 200 ms (i.e., 5 Hz). In cTBS, a train of TBS was delivered without interruption for 40 s (for a total of 600 pulses). In imTBS, a 5 seconds train of TBS was repeated every 15 seconds for a total of 600 pulses. To comply with the original study of Huang *et al*.^38^ and safety standards^43^, the stimulus intensity was set at 80% of the active motor threshold (aMT) (mean of maximal stimulator output: 43.1 ± 4.7% in the first day and 43.6 ± 6.8% in second day). The aMT is defined as the minimum stimulation intensity that can elicit MEPs larger than 200 µV in at least 5 of 10 successive trials during a grip-force measurement with 20% of the maximum force of the contralateral hand. The angle of the coil was kept perpendicular to the underlying gyrus with the handle pointing upward and supported manually. No particular discomfort or other negative side-effects were reported.

#### spTMS and electromyographic recordings

Fast and focal measurements of corticospinal excitability (MEPs) were obtained during EB by using spTMS while participants observed the model’s right index finger abductions. spTMS was randomly delivered between 300 and 500 ms from the onset of the GO signal (see above) by a 70 mm figure-of-eight stimulation coil connected to a Magstim 200 magnetic stimulator. The coil was positioned tangentially over participants’ left primary motor cortex with the handle oriented backward and laterally 45° away from the midline approximately perpendicular to the central sulcus. For each action observation trial (N = 20), we simultaneously recorded spTMS-elicited MEPs from the participants’ abductors of the right index (first dorsal interosseous; FDI) and of the right little (abductor digiti minimi; ADM) fingers by using self-adhesive disposable Ag–AgCl surface electrodes. A ground 1.5 cm metal electrode was placed on the dorsal surface of the wrist. The electrodes were on both the model’s and participant’s hands but the signal was recorded only from the participant’s muscles. The EMG was amplified 1000 times, digitized at 5 kHz, and band-pass filtered (between 10 and 1000 Hz) with a mains hum notch filter at 50 Hz. The optimal scalp position from which MEPs with maximal amplitude were elicited in both resting FDI and ADM muscles was detected by moving the coil over the left motor cortex while delivering TMS pulses at constant intensity. The stimulus intensity employed for spTMS stimulation was set at 120% of each subject’s resting motor threshold (rMT) (mean of maximal stimulator output: rMT 75.7 ± 7.9% in the first day and 75.2 ± 8.1 in the 2nd day). The rMT is defined as the lowest stimulator output that evokes at least five of ten successive MEPs with amplitude greater than 50 μV in the relaxed FDI and ADM muscles.

### Analysis

The Skewness and Kurtosis values of the data (except the Kurtosis of the proprioceptive drift in the cTBS Synchronous condition in EB) were within the limits of ± 2 considered acceptable in order to prove normal univariate distribution^53^.

#### Behavioural data

To assess the proprioceptive drift, we first computed the difference between the objective and the subjective localisation of the participant’s index finger. Then, we subtracted the measure obtained before from that obtained after each condition to obtain the proprioceptive drift. The conditions were the following: TBS (cTBS and imTBS), BLOCK (IB and EB) and STROKING (Synchronous/Self and Asynchronous/Other). The resulting data were submitted to a non-parametric Friedman ANOVA.

The mean agreement ratings to each of the first three statements in the RHI questionnaire were entered into a multivariate analysis of variances (MANOVA) with the within-participants variable TBS and STROKING, BLOCK and the three questionnaire items assessing the experience of ownership over the model’s hand as dependent variables. We run a further repeated measure ANOVA on the agency question (statement 4) with the within-participants factors TBS and STROKING.

#### Neurophysiological data

Individual peak-to-peak MEP amplitudes were calculated as the absolute distance between the minimum and maximum values observed within a search window starting at 10 msec and ending at 80 msec after the TMS pulse. We discarded outliers (with values exceeding 2.5 standard deviations from the mean values of each participant, muscle and TBS condition^12^) for both the background EMG activity (in the 100 ms preceding the TMS pulse) and the MEP amplitudes: 4.4% of trials were excluded in the FDI and 4.3% in the ADM. The remaining MEP values were normalised by calculating the natural logarithm of the facilitation ratio (MEP amplitudes recorded from the FDI / MEP amplitudes recorded from the ADM) and outliers were excluded (1%). The overall number of excluded trials did not differ across conditions (oneway ANOVA F < 1; p = 0.6). The average number of trials in each condition was 17.9 ± 1.7 (mean number of trials ± standard deviation), with a range from 13 to 20.

The data were analysed using linear mixed models^54^ implemented in the lme4 package^55^ in R^56^. We used deviation coding for each of our fixed effects. Our model included the fixed effects STROKING and TBS, and the interaction between them. The random effect in our model was participant id, with maximal random intercepts and slopes^57^. Restricted maximum likelihood estimation was used to generate the model parameters. Degrees of freedom for the t-statistics were approximated using the Satterthwaite method. Follow up tests on the significant interaction effect were conducted using the emmeans package^58^ with Bonferroni corrected p-values and the Kenward-Roger method to approximate degrees of freedom.

## Results

### Behavioural data

The analysis on the proprioceptive measures revealed no significant differences [χ^2^(7) = 4.64, p = 0.7].

**Figure 2:**
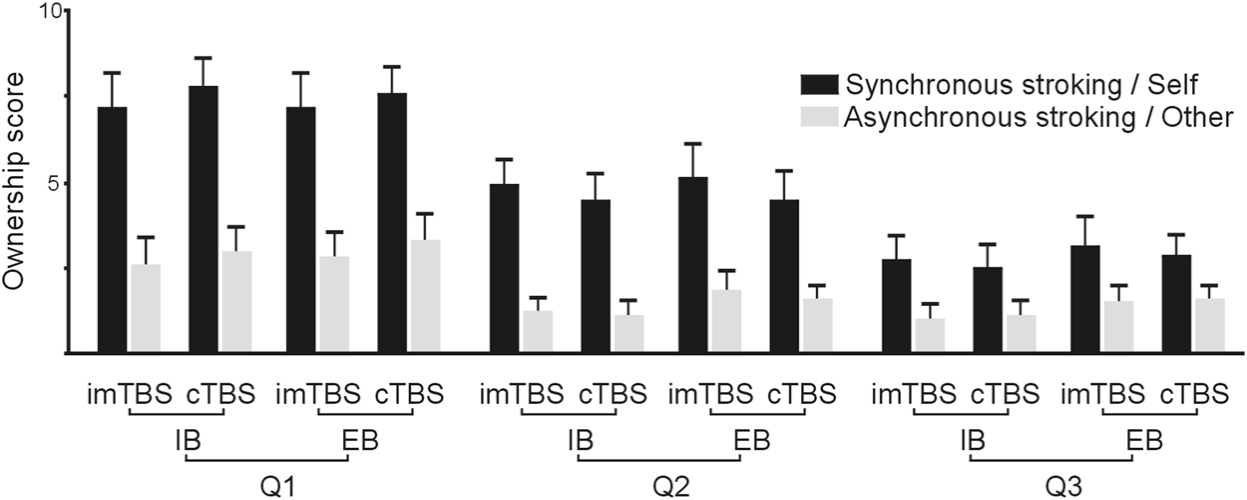
Means and standard errors of the RHI questionnaire assessing the ownership of the observed hand in the different experimental conditions.

The analysis on the three statements of the questionnaire assessing ownership showed a significant main effect for STROKING [F(3, 118) = 30.21, p < 0.001; Fig. 2] indicating that mean values expressed for the Synchronous/Self condition where always larger than those of the Asynchronous/Other condition. Other effects were not significant (all p > 0.5). Therefore, as expected, the synchronous stimulation generated the illusion of ownership over the model hand more than the asynchronous stimulation. Moreover, the results showed that TBS did not interfere with the results obtained from the ownership questionnaire.

No effects or interactions were significant (all p > 0.05) in the analysis on the agency question (statement 4). Mean values expressed for the Synchronous/Self condition were only numerically larger than those of the Asynchronous/Other condition.

### Agent-specific representations in the motor system

**Figure 3:**
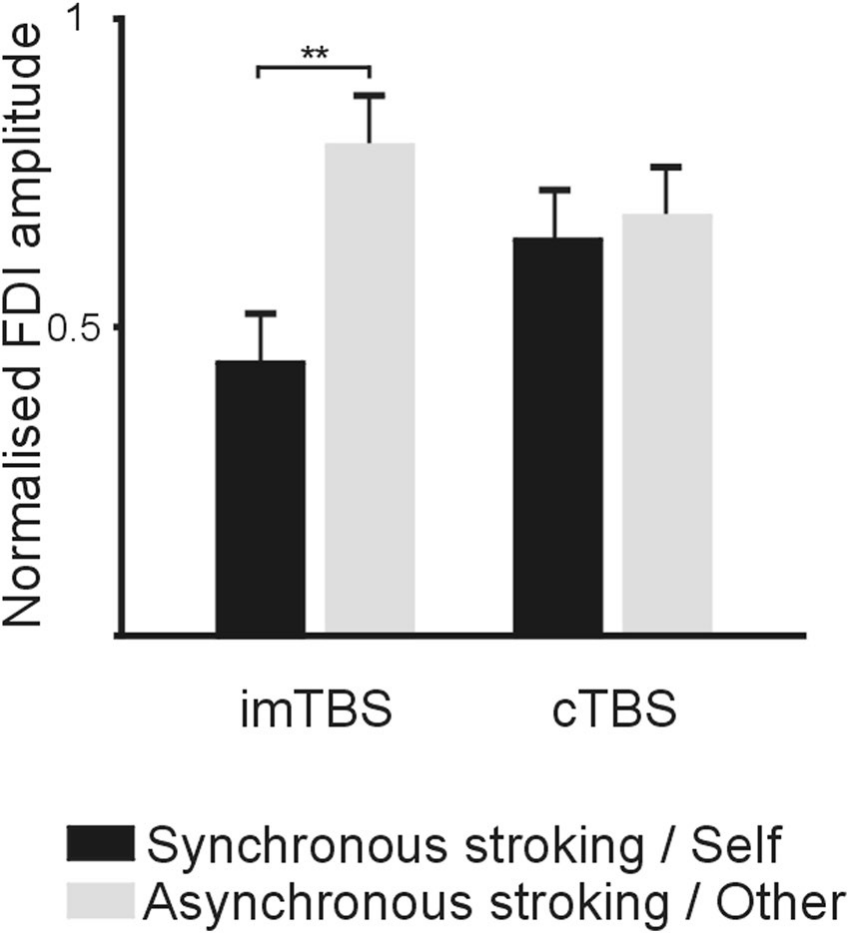
Means and standard errors of normalised MEP amplitudes recorded during the observation of right index finger’s abductions in the four experimental conditions of the Experimental Block. MEPs are express as a facilitation ratio over those recorded from the little (i.e., control) finger.

The main effect of STROKING was statistically significant (p = 0.01; Table 2), with MEPs for Asynchronous/Other generally larger than those recorded in the Synchronous/Self condition, whilst the main effects of TBS was not (p > 0.05). Importantly, a significant interaction between TBS and STROKING (p < 0.05) revealed that in the control imTBS condition the mean normalised MEP values were significantly larger (p < 0.01, Bonferroni corrected) in the Asynchronous/Other condition (0.76 ± 0.06; mean ± SE) than in the Synchronous/Self one (0.44 ± 0.07; Fig. 3). In contrast, mean normalised MEPs recorded after cTBS did not differ between the two stroking conditions (p = 1; Synchronous/Self = 0.56 ± 0.07; Asynchronous/Other = 0.68 ± 0.06). Other comparisons were not statistically significant (all p > 0.9).

**Table 2:** Results of the Linear Mixed Model.

	Estimate	SE	df	t	p
Intercept	**0**.**62**	**0**.**21**	**15**	**2**.**99**	**0**.**009**
Stroking	**0**.**19**	**0**.**06**	**14**.**81**	**2**.**95**	**0**.**01**
TBS	0.04	0.15	14.95	0.27	0.79
Stroking * TBS	**0**.**3**	**0**.**14**	**14**.**93**	**2**.**18**	**0**.**046**

Significant effects are highlighted in bold.

## Discussion

Without an accurate sense of agency, or the feeling of control over intended self-generated movements^59^, people are unable to successfully recognise ‘who’ is the author of an observed action and may attribute movements executed by others to themselves^17^. Obviously, such condition may have dramatic social effects such as compromised interaction and cooperation with other individuals^60–64^.

A great deal is known about the brain structures associated with the feeling of agency at a higher-order, conscious level.Over the years, brain imaging^34,65–67^ and repetitive TMS^68^ studies have consistently confirmed the role of the IPL in carrying out neural computations associated with the awareness of initiating a movement or with attributing an action to its correct agent^16–20,35^ (for a review, see^21,22^). This evidence from the healthy brain was also confirmed by studies carried out on brain-damaged patients: indeed, patients with IPL lesions may become aware of their movements only after having executed them^17^ or may perform movements without any conscious intention^69^. In agreement with these investigations, a particularly striking intracranial stimulation experiment^22^, conducted during awake brain surgery, showed that conscious feelings of having performed a movement, in the absence of any muscle contraction, can be evoked by high intensity stimulation of the left and right IPL. An opposite pattern was observed when stimulating the premotor cortex, which resulted in the induction of overt motor responses of which the patients remained unaware. Further evidence could be found in patients suffering from schizophrenia, as hyper-activation of this area can determine abnormal attribution of intentions^23^.

Having determined which is the neural correlate of the perceived agency at a higher-order and reflective level, the question arises as to whether the IPL is also well suited to shape the pre-reflective experience of action authorship in the sensorimotor system. The evidence gathered so far indicates that it may emerge from the predictions, generated in the posterior parietal cortex, that the brain makes about the movement before action onset^21^. This model suggests that an efference copy of a motor command is sent towards the parietal cortex during movement execution^70,71^ where the prediction of the intended action is tested against the sensory and proprioceptive feedback^1,27–29^. This comparator mechanism should allow the detection, at a sensorimotor level, as to whether an action is self- or other-generated. Some research further suggests that these low-level agent-specific signals in the action system^12,13,72^ may support the explicit representation thereof ^73^. Nonetheless, the former were only characterised in the primary motor pathway and the question of which neurophysiological mechanism shapes action attribution at the sensorimotor level remained largely unanswered. In the present work, we were able to confirm the causal involvement of the IPL in action authorship by demonstrating that interference with IPL-M1 functional interaction resulted in the disruption of self-other differentiation in the action system as already identified by Schütz-Bosbach and colleagues^12,13^. In particular, cTBS seemed to selectively interfere with self-related representations: a numerical facilitation (instead of an inhibition) of MEPs was recorded in the condition in which participants observed self-attributed actions (during synchronous stroking). This could be interpreted as evidence that cTBS reduced the inhibition that the IPL exerts over the M1^37^, which in turn may drive the intra-cortical inhibition and reduced facilitation measured in control conditions during the observation of self-related actions^13^. It is worth mentioning that our conclusion of a causal involvement of the left IPL in self-owned hand movements is consistent with a recent EEG experiment^36^ showing that transcranial alternating current stimulation of the left IPL reduced the activation of motor-related processes during the observation of hand movements from an egocentric (but not allocentric) viewpoint.

Our result triggers some other questions, such as what are the known effects of cTBS and how they could have influenced action attribution? Compared to classical repetitive TMS and other protocols available (for a review, see^74^), the cTBS approach induces prolonged neural inhibition at stimulated loci (>45 minutes^38,75^;) after only a short (40 sec) and low intensity stimulation consisting of 600 stimuli. At stimulation intensity of 80% of the active motor threshold, cTBS produces a decrease of intracortical excitability^51^ that has been associated with long-term depression (LTD)-like mechanisms^38,76^. Accordingly, it appears reasonable that cTBS of IPL could drop the firing of the excitatory parietal-motor projections (non-local cortico-cortical projection are glutamatergic) originating from the IPL, which are known to inhibit the ipsilateral M1^37^ through the ventral premotor cortex^77^. Indeed, anatomical studies, DTI tractography and analysis of the strength of connectivity^77–81^ reveal that the IPL is extensively connected with the ventral premotor cortex by different bundles of the superior longitudinal fasciculus, whilst only sparse connections are found with M1. In turn, the ventral premotor cortex plays as a relay in the parietal-to-motor network connecting IPL to M1. As a matter of fact, cTBS to this area disrupts the parietal-motor connectivity^77^.

The recent work by Karabanov and colleagues^82^ further demonstrates that the parietal-to-motor inhibition, which is observed at rest^37^, is preserved when people experience ownership over a moving rubber hand but not when they lack this illusory experience. This is consistent with the present findings for both TBS paradigms. While the mean MEP values for the Synchronous/Self condition were significantly lower than in the Asynchronous/Other after imTBS, cTBS of the IPL impaired agent differentiation in the motor system as indicated by a lack of a difference between the mean MEPs for the two condition. More precisely, cTBS seemed to selectively facilitate the illusorily self-attributed actions, when compared to the homologous condition in imTBS. We could therefore argue that cTBS of IPL affected the GABAergic inhibitory circuits, at the level of local interneurons within the primary motor cortex^13,83,84^, as well as the reduced facilitation associated with actions attributed to the self^12,13^. We believe that our approach demonstrates the IPL’s role in differentiating the self from the other at the sensorimotor level and provides reliable evidence of the causal functional interactions between the IPL and M1 in action authorship, although we cannot substantiate any information about the anatomical pathways that might mediate it.

It is worth noting, however, that IPL stimulation had no impact on the successful induction of the RHI, as assessed from the first three statements of the questionnaire, indicating that the stimulation did not affect the experience of ownership over the model’s limb. Moreover, we found that the ratings regarding the experience of agency (statement 4) did neither differ across the experimental conditions nor across the pre-post comparison, suggesting that our modified version of the RHI paradigm did not induce illusionary experience of control (i.e., agency) over the observed movements in a passive observer but impact the experience of owner- and authorship (i.e. self-other attribution) of the observed action only (for the conceptual distinction between agency and ownership see e.g.^85,86^.

However, it is important to note that the present study does unfortunately not allow to draw any strong conclusions about the potential relation between the differential response pattern at the level of the primary motor cortex and the phenomenological experience of hand/action authorship as assessed by the Rubber-hand illusion questionnaire, since we assessed illusionary experiences only once after each experimental block. Future studies should therefore consider to use a trial-by-trial assessment of illusionary experience to predict corticospinal excitability, respectively and vice versa^73^ and in this way further proof the validity of the neurophysiological response as a proxy of explicit sensations of movements and judgements of self-other attribution.

As far as the proprioceptive drift is concerned, we found no statistically significant results. Its absence may be due to TMS-induced muscle twitches, necessarily induced in an MEP study, that have already been shown to affect the drift measure (for similar arguments see^12^). Another explanation may be related to a cTBS-induced disruption of visuo-tactile integration^87^ that may have modulated the perceptual drift while leaving the subjective ownership ratings intact. Such an occurrence has been shown on various occasions previously^88–90^. Failure of recalibrating the limb in the RHI was also associated with motor symptoms in dystonia, in which the experience of the illusion was again retained^91^. This points to the direction of a failure in the integration of visual-tactile input with the proprioceptive information in order to update the body part position. In other words, one could argue that cTBS introduced a conflict that affected the perceived synchronicity of brush-stroking of the participant’s and the model’s fingers during the RHI. Whether this interfered with the sensorimotor representation of self- and other-related actions, while leaving the ownership feeling intact, is unclear. Reduced drift has also been reported in autism spectrum disorder when individuals were involved in a grasping task under the effects of the RHI^92^. In this work, Paton and colleagues^92^ observed that healthy subjects were facilitated in their action when they were in the other condition compared to the self one. In patients, the pattern was reversed: the authors measured a facilitation for the self condition, probably due to a self-oriented behaviour (less representation of the other). Interesting, individuals with autism also display atypical patterns of corticospinal excitability during action observation^93^, such as lower modulation of M1 excitability^94^, reduced MEP facilitation^95^ and cortical inhibition deficits^96^. This atypical patterns of self–other representations may lead to reduced social abilities^97^ and may depend on anomalies of the IPL, such as reduced thickness that was associated to this disorder^98^.

In conclusion, we argue that changes in parietal-motor connectivity modulate action authorship at the sensorimotor level. On the one hand, our work gives further experimental support to models of action authorship as emerging from neural computations carried out in the parietal cortex^19,99^. On the other hand, it identifies the IPL as the provider of core information necessary for social differentiation at the sensorimotor level. Future studies will need to better characterise this neuropsychological processing and explore its impact on fronto-parietal deficits such as with schizophrenia and autism^100^.

## Data Availability

All data are made available by authors upon request.
